# FXR and NASH: an avenue for tissue-specific regulation

**DOI:** 10.1097/HC9.0000000000000127

**Published:** 2023-04-14

**Authors:** Zakiyah Henry, Vik Meadows, Grace L. Guo

**Affiliations:** 1 Department of Pharmacology and Toxicology, Rutgers University, Piscataway, New Jersey, USA; 2 Environmental and Occupational Health Science Institute, Rutgers University, Piscataway, New Jersey, USA; 3 Department of Veterans Affairs New Jersey Health Care System, East Orange, New Jersey, USA

## Abstract

NASH is within the spectrum of NAFLD, a liver condition encompassing liver steatosis, inflammation, hepatocyte injury, and fibrosis. The prevalence of NASH-induced cirrhosis is rapidly rising and has become the leading indicator for liver transplantation in the US. There is no Food and Drug Administration (FDA)-approved pharmacological intervention for NASH. The farnesoid X receptor (FXR) is essential in regulating bile acid homeostasis, and dysregulation of bile acids has been implicated in the pathogenesis of NASH. As a result, modulators of FXR that show desirable effects in mitigating key characteristics of NASH have been developed as promising therapeutic approaches. However, global FXR activation causes adverse effects such as cholesterol homeostasis imbalance and pruritus. The development of targeted FXR modulation is necessary for ideal NASH therapeutics, but information regarding tissue-specific and cell-specific FXR functionality is limited. In this review, we highlight FXR activation in the regulation of bile acid homeostasis and NASH development, examine the current literature on tissue-specific regulation of nuclear receptors, and speculate on how FXR regulation will be beneficial in the treatment of NASH.

## Graphical abstract

**Figure:**
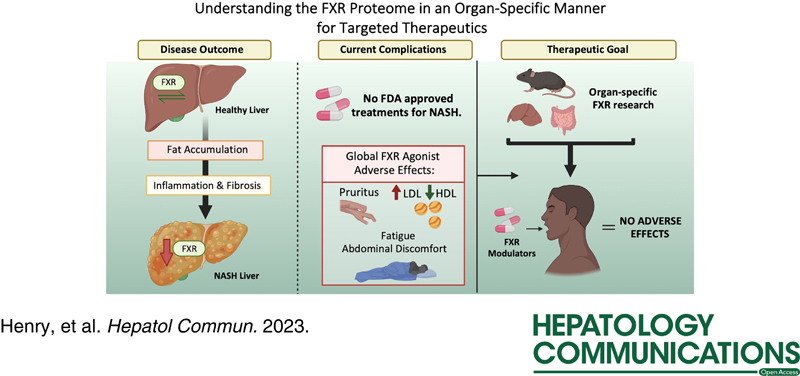

## INTRODUCTION

NAFLD is the most common chronic liver condition in the US, with an estimated 25% of US adults suffering from this disease, particularly simple fatty liver. NAFLD encompasses a spectrum of liver conditions characterized by fat accumulation in the liver, not caused by excessive alcohol consumption, which may develop into NASH. Approximately 20%–25% of the NAFLD population have NASH (5% of US adults). NASH is characterized by hepatocyte ballooning, inflammation, and varying degrees of fibrosis, in addition to steatosis. Excessive fibrosis can lead to cirrhosis, an end-stage liver disease, and increase the risk of HCC.^1^,^2^ NASH-induced cirrhosis has become the leading indicator for liver transplantation in the country, and its prevalence is rapidly rising.^3^–^5^ The progression from simple fatty liver to NASH is not well elucidated, and although the pathogenesis of NASH has been speculated and theorized, its etiology has yet to be confirmed.^6^ The current recommended treatment of NASH is lifestyle modification such as diet and exercise with no FDA-approved pharmacologic interventions.

**FIGURE 1:**
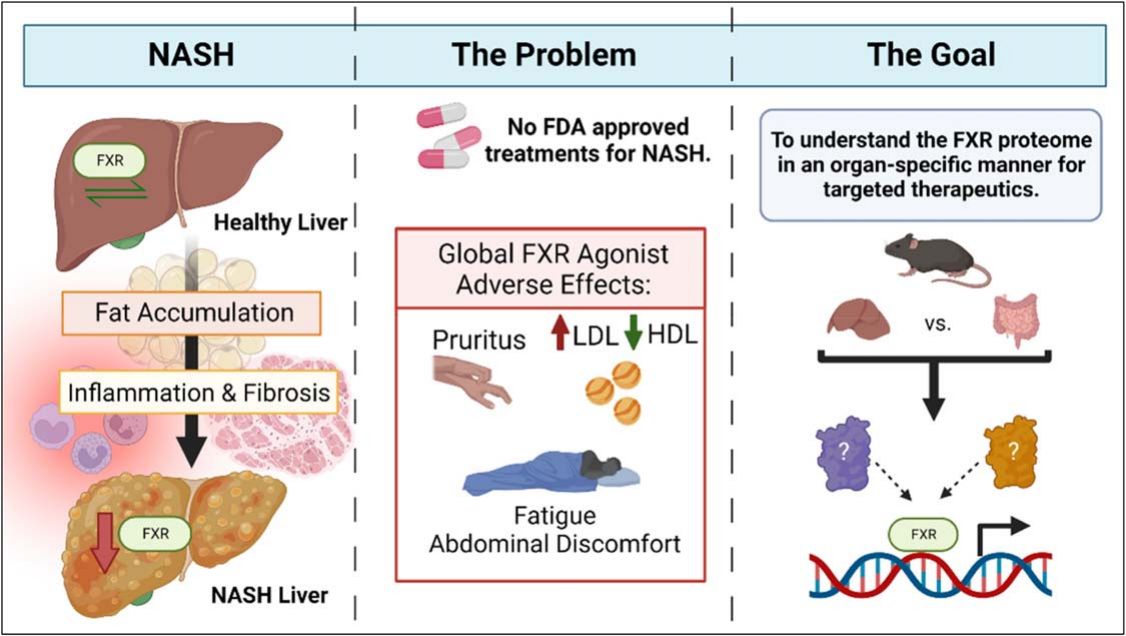
Overall significance. NASH develops following fat accumulation and subsequent hepatic inflammation and scarring. There are currently no approved pharmaceutical therapeutics for NASH patients, and global FXR agonists, currently undergoing clinical trials, demonstrate debilitating adverse effects like pruritus, abnormal cholesterol levels (elevated LDL and decreased HDL), fatigue, and abdominal discomfort. Because of visualized varied FXR functions, it is critical to understand how FXR activation affects NASH development in an organ-specific manner to identify proper therapeutic targets. Abbreviations: FDA, Food and Drug Administration; FXR, farnesoid X receptor.

A ligand-activated transcription factor (TF) and type II nuclear receptor (NR), farnesoid X receptor (FXR), has been identified as a clinical target for therapeutic intervention for NASH and other chronic liver diseases. FXR has a wide range of functions that are beneficial in the treatment of NASH, including the reduction of steatosis, inflammation, and fibrosis, through the transcriptional activation and/or suppression of various biological pathways.^7^ Synthetic steroidal or nonsteroidal agonists of FXR have been developed for the treatment of NASH and are currently undergoing clinical trials, such as obeticholic acid, cilofexor, nidufexor, and tropifexor.^8^,^9^ The current FXR agonists activate whole-body FXR and display favorable effects in the treatment of NAFLD/NASH^10^–^12^; however, adverse side effects such as pruritus, cholesterol homeostasis imbalance (increases in LDLs and decreases in HDLs), fatigue, and abdominal discomfort have been reported in patients with NASH and other chronic liver diseases after treatment with FXR agonists.^9^,^13^ There is an urgent need to determine the tissue-specific role(s) of FXR to prevent adverse effects and to develop targeted and efficacious therapies for NASH patients (Figure 1). This review examines factors that contribute to FXR tissue-specific modulation and their potential effect on the therapeutic development for NASH.

### FXR

#### Introduction to NRs

NRs are a family of ligand-activated TFs that regulate various biological processes and functions. There are over 500 members of this superfamily that are further divided into 7 subfamilies or subclasses: NR1 (thyroid hormone-like), NR2 (HNF4-like), NR3 (estrogen-like), NR4 (nerve growth factor IB-like), NR5 (fushi tarazu-F1-like), NR6 (germ cell nuclear factor-like), and NR0 (which do not contain a DNA binding domain).^14^,^15^ FXR is an adopted orphan NR1 that is activated by bile acids (BAs). Specifically, the FXR gene (*Nr1h4*) was first cloned in 1995 from mouse and rat liver,^16^ and BAs were discovered to be endogenous ligands of FXR in 1999.^17^ FXR is highly expressed in several organs and cell types, including hepatocytes, kidneys, adrenal glands, enterocytes, and to a lesser extent, HSCs, cholangiocytes, white adipose tissue, and immune cells.^18^ Six isoforms of FXR have been discovered (FXRα1-4 and FXRβ1-2), with FXRα being greatly expressed in the liver, distal small intestine (ileum), and adrenal glands, and FXRβ in the colon, proximal small intestine (duodenum), and kidney in humans.^19^–^21^

#### FXR and BA regulation

BAs are amphipathic molecules essential in the absorption of dietary fats, cholesterol, and lipid-soluble vitamins (vitamins A, D, E, and K). They are synthesized by hepatocytes through complex and tightly regulated processes involving at least 17 different enzymes^22^ through 2 major pathways as a result of cholesterol catabolism, the classical and alternative pathways. The classical, also known as the neutral pathway, is initiated with the rate-limiting enzyme cholesterol-7α-hydroxylase (CYP7A1), followed by sterol 12α-hydroxylase (CYP8B1) to yield cholic acid (CA), whereas the alternative, or acidic pathway, consists of sterol-27-hydroxylase (CYP27A1) and 25-hydroxycholesterol 7-alpha-hydroxylase (CYP7B1) to make chenodeoxycholic acid (CDCA).^23^,^24^ In mice, CDCA is converted to β-muricholic acid by CYP2C70, which is more hydrophilic and regarded as a strong FXR antagonist.^25^,^26^

CA and CDCA are produced in the liver, where they are conjugated with glycine (mainly in humans) or taurine (mainly in mice), which decreases their initial hydrophobicity and increases solubility. Once conjugated, the bile salts are effluxed out of hepatocytes by the bile salt export pump (BSEP) and multidrug resistance-associated protein 2 (MRP2) into the bile canaliculi to be excreted out of the liver through bile ducts. Cholangiocytes, epithelial cells of the bile duct, modify bile salts by diluting and alkalizing bile through bicarbonate or other secreted compounds.^27^ Bile is stored in the gallbladder in most species until stimulated for release by cholecystokinin, postprandial, into the duodenum through the Sphincter of Oddi for emulsification, digestion, and absorption of lipids in the small intestinal tract. The primary BAs that make up the human BA pool are CDCA and CA, which are converted to secondary BAs, lithocholic acid and deoxycholic acid, respectively, in the gut due to microbial modification.^28^ BAs also affect the gut microbiota composition, which can in turn alter the BA species pool through a variety of modifications, including deconjugation, dehydroxylation at carbon 7, and oxidation and epimerization, increasing BA diversity.^29^ Bacteria expressing the bile salt hydrolase gene can cleave glycine and taurine^24^ from conjugated BAs, and a complex of bacterial enzymes encoded by the Bai operon can further modify BAs into secondary structures that are not toxic to the microbiota population.^29^,^30^ Approximately 95% of BAs are reabsorbed in the terminal ileum into enterohepatic circulation through ileal apical sodium-dependent BA transporter (ASBT) and organic solute transporter alpha and beta (OSTα/β).^31^,^32^ Circulated BAs enter the liver through hepatic Na^+^/taurocholate cotransporting polypeptide (NTCP) for conjugated BAs or by organic anion-transporting polypeptide (OATP) for unconjugated BAs. The remaining 5% of BAs can be deconjugated by gut microbes and passively absorbed by the colon (like deoxycholic acid) or excreted from the body through the feces (mainly lithocholic acid).^33^ Secondary BAs are, in general, more hydrophobic and toxic than primary BAs.^34^

BAs not only function as critical components of digestion but also as powerful signaling molecules and endogenous ligands of several NRs, including pregnane X receptor and vitamin D receptor, in addition to activating FXR.^35^ CDCA is the most potent BA activator of FXR, followed by CA, deoxycholic acid, and lithocholic acid.^33^,^36^,^37^ FXR is the main regulator of BA homeostasis and is especially important in activating negative feedback inhibition mechanisms, such as ileal FGF15 in mice, FGF19 in humans, and hepatic small heterodimer partner (SHP) to suppress BA synthesis.

#### Tissue-specific role of FXR in regulating BA homeostasis

Because of amphipathic chemical properties, BAs behave as detergents and, if not properly regulated, can induce liver injury, inflammation, hepatocyte apoptosis, and cholestasis.^38^–^41^ Extensive or chronic liver damage can lead to cholestasis and even malignancy development in patients, making the regulation of BA synthesis a key topic in the field of hepatology.^42^ FXR is expressed in various organs and cell types such as the pancreas, lungs, kidneys, liver (hepatocytes, cholangiocytes, and stellate cells), and intestine (enterocytes).^43^–^47^ FXR function is largely understood in hepatocytes and ileal enterocytes, but its role in other cell types is not fully understood.

Intestinal FXR, specifically in the ileum, is the main regulator of BA synthesis by means of the FXR-FGF15/19 pathway that operates by mechanisms of negative feedback inhibition.^48^ FXR’s activation in the ileum induces FGF15 secretion in mice^49^ and FGF19 in humans,^50^ into the portal vein to the liver where it binds and activates its receptor, FGF receptor 4 along with β-Klotho, in hepatocytes to activate mitogen-activated protein kinase signaling pathways.^51^,^52^ FGF receptor 4 activation signaling inhibits the gene expression of *Cyp7a1*, suppressing the classical pathway of BA synthesis. Intestinal FXR controls BA synthesis, mainly at night, through high *Fgf15* expression in the intestine^53^; however, hepatic FXR regulates BA synthesis through induced expression of *Shp*, which binds to liver receptor homolog 1 inhibiting *Cyp8b1* gene transcription and minorly *Cyp7a1*.^51^ Hepatocyte FXR activation also induces the expression of BA efflux transporters, such as *Bsep* and *Ostα/β*, in the liver to promote enterohepatic BA circulation and prevent cholestasis.^54^,^55^ Because of the lack of FXR specificity for primary and secondary BAs and BA dose-dependent cellular toxicity, the generation of synthetic ligands for FXR activation has been of increased interest.

### Pioneer Factors

#### Regulators of transcription

With genome-based studies becoming especially critical in the study of NASH and other chronic liver diseases, understanding gene expression in an organ-specific manner may hold the key to identifying ideal therapeutic targets. Pioneer factors (PFs), a subset of TFs recognized as the proteins capable of binding condensed chromatin to regulate transcription in a cell-specific manner,^56^ have recently been recognized as an avenue for chronic liver disease research. Through this process, PFs, and their dynamic expression, implement cell fate and organ development^56^; however, it has been recently proposed that chromatin opening, and subsequent expression of silent genes, is mediated by PFs and non-PFs alike.^57^ To target these silent areas in the genome, PFs must recognize their target DNA sequences on the nucleosome.^58^ Despite these controversies, understanding the organ-specific role of TFs remains a key area of study for drug development.

Forkhead Box A (FOXA) is a family of TFs vital in foregut endoderm for hepatic differentiation and induction of liver-specific genes such as *albumin*.^59^ The DNA binding domain of FOXA (“winged helix” structure) resembles the nucleosomal binding domain of the linker histone, causing its displacement and opening of the chromatin.^60^ The winged helix structure, also known as the forkhead domain, is highly conserved in each isoform. In the liver, FOXA1 and FOXA2^61^ are required for early organ induction,^56^,^62^ with FOXA2 deletion being embryonically lethal, whereas another set of PFs known as GATA-binding proteins, specifically GATA-4 and GATA-6,^63^,^64^ is redundantly expressed and required for the early organ development from the foregut endoderm. Conditional triple-knockout of *Foxa1/2/3* in hepatocytes of adult mice resulted in eventual liver failure 15–20 days after deletion.^65^ In these mice, hepatocyte nuclear factor 4 alpha (HNF4α) was continually expressed, but there was minimal chromatin accessibility at FOXA-HNF4α cobound sites, confirming that FOXA chromatin manipulation is necessary for adult liver function.^65^ It has been found that overexpression of GATA-6 in patients with NAFLD resulted in increased HSC activation and subsequent fibrosis.^66^ Interestingly, hepatocyte-specific deletion of GATA-4, through the albumin promoter, resulted in increased steatosis and insulin resistance in a murine model fed high-fat diet.^67^ These studies demonstrate the complexity of PFs postdevelopment and highlight an important role for them in the development and progression of steatosis and NASH. There are limited studies investigating PF function in NASH; however, the proteome created by PF and TF binding may provide the key to organ-specific therapeutic targeting.

#### FOXA2 and BA homeostasis

**FIGURE 2:**
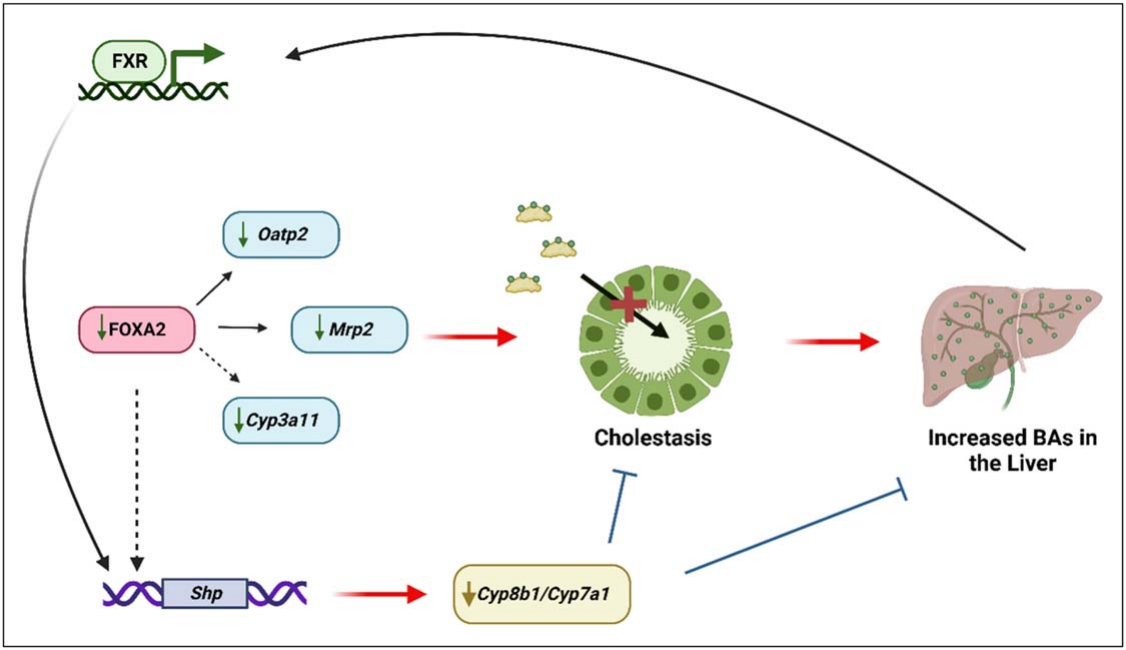
FOXA2 and BA homeostasis. In the liver, FOXA2 directly regulates the expression of genes of transporters *Oatp2* and *Mrp2* involved in BA transport and, indirectly, *Cyp3a11*. Deficiency of FOXA2, as seen in cholestatic patients, has decreased the gene expression of *Oatp2, Mrp2* (along with *Mrp3* and *Mrp4*, not shown), and *Cyp3a11*, contributing to cholestasis and increased BA accumulation within the liver. Excess BAs activate FXR, which in turn induces gene expression of *Shp* to suppress the expression of genes encoding rate-limiting enzymes for BA synthesis, *Cyp7a1* and *Cyp8b1*, to mitigate cholestasis and excess BAs in the liver. FOXA2 is also believed to bind alongside FXR in the upstream regulatory region to elicit these effects. Abbreviations: BA, bile acid; FXR, farnesoid X receptor.

FOXA2 (previously known as HNF-3β) is essential for murine liver development and remains critical in the adult liver for BA, glucose, and lipid homeostasis.^68^–^71^ *Foxa2*-deficient mice display an accumulation of BAs in the liver (Figure 2).^72^ Furthermore, FOXA2 has been shown to regulate hyperbilirubinemia in mice and patients with sepsis and acute liver failure by the upregulation of MRP2.^73^ FOXA2 directly and indirectly regulates the gene expression of hepatic transporters, *Oatp2*, *Mrp2*, *Mrp3*, and *Mrp4*, and indirectly regulates *Cyp3a11* that encodes a key P450 phase I enzyme, contributing to BA accumulation.^69^,^73^ Chromatin immunoprecipitation (ChIP) conducted with an anti-FOXA2 antibody on livers with no FOXA2 suggests that *Mrp2* and *Oatp2* genes are direct targets of FOXA2 *in vivo*. FOXA2 replaces FXR to maintain the expression of *Mrp2* in patients with acute liver failure excluding sepsis.^73^ In fact, mice with hepatocyte-specific *Foxa2* ablation displayed reduced *Cyp7a1*, *Cyp7b1*, *Cyp8b1*, *Cyp27a1*, and *Ntcp* gene expression following standard diet feeding, insinuating a key role for *Foxa2* in BA regulation.^69^ It has also been shown that pediatric and adult cholestatic patients have reduced hepatic *FOXA2* expression, further exemplifying its importance in liver disease progression.^69^ FOXA2 and FOXA1 also regulate bile duct and gallbladder development by manipulating chromatin accessibility for glucocorticoid receptor binding.^74^,^75^

**FIGURE 3:**
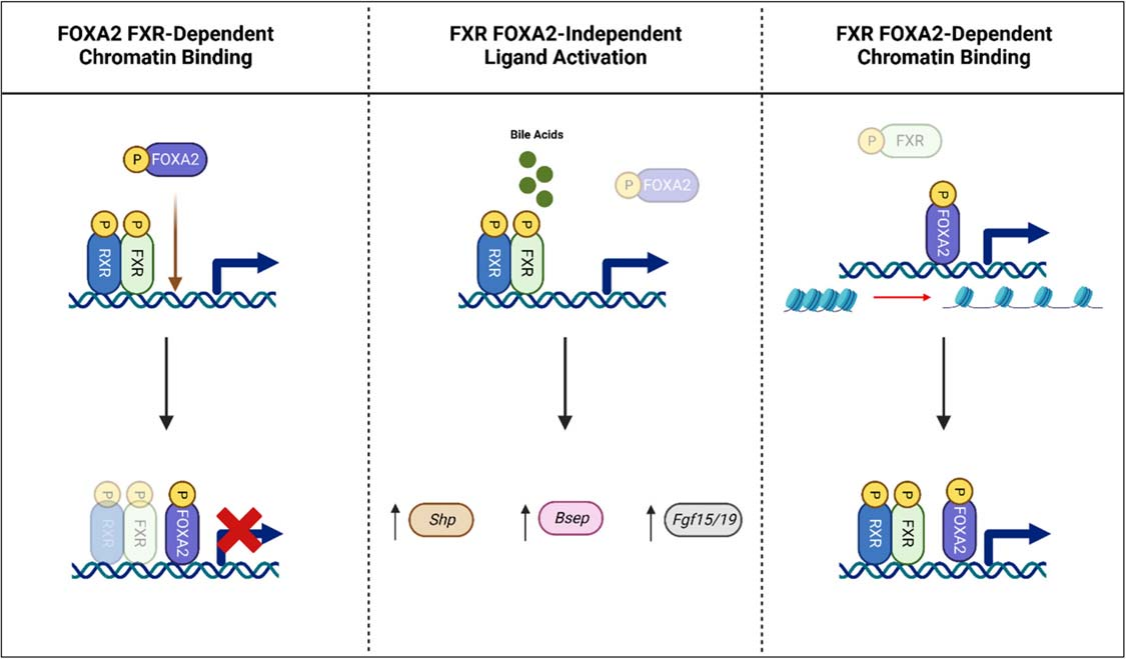
Proposed FXR and FOXA2 interactions. Three proposed FXR and FOXA2 interactions: (1) FOXA2 binding is dependent on FXR. In addition, when bound, FOXA2 may repress FXR (which heterodimerizes with RXR) transcriptional activity. (2) Activation of FXR by ligand-binding (such as BAs) is independent of FOXA2, and activation of FXR leads to transcriptional regulation of various genes such as *Shp*, *Bsep*, and *Fgf15/19*. (3) FXR chromatin binding and activation is dependent on the presence of FOXA2, which displaces histones in highly condensed areas of chromatin for a more open configuration to allow FXR binding. Abbreviations: FOXA1/2, forkhead box A1/2; FXR, farnesoid X receptor.

It is possible that BA activation of FXR may acutely activate the transcription of *Foxa2*.^76^ In mice, FXR and FOXA2 bind the upstream regulatory region of *Shp*, with *Shp* induction decreasing BA production by downregulation of *Cyp7a1* transcription (Figure 2).^77^ The field remains controversial on the actual interactions of FXR and FOXA2. There are several proposed interactions. It is believed that the binding of FOXA2 is dependent on FXR, and FOXA2 may repress FXR transcriptional activity on several genes, including *Shp*, by a tethering mechanism.^78^ This proposed mechanism would explain how FOXA2 could regulate FXR tissue-specific functionality. Contrarily, it has been suggested that FXR ligand–directed activation remains FOXA2-independent while its chromatin binding is FOXA2 dependent^76^,^79^; however, it has also been shown that FOXA2 is required for ligand-bound FXR DNA binding and activation.^78^,^79^ Similarly, FOXA2 occupancy is increased dramatically when FXR is bound by an agonist, leading to the belief that FOXA2 is not bound to DNA before FXR ligand activation. FOXA2 evicts nucleosomes allowing for the opening of chromatin for FXR-binding accessibility and increased transcription. However, FOXA2 is believed to repress the transcriptional activity of FXR appropriate for the maintenance of a particular physiological state. These works suggest an interdependent relationship between FOXA2 and FXR DNA binding during ligand activation (Figure 3). The mechanism of interaction between FXR and FOXA2 is not well understood, and further studies may allow for a deeper understanding of their complex interactions in health and disease.

### Importance of FXR tissue specificity in the treatment of NAFLD/NASH

#### Implications of BAs in NAFLD/NASH progression

Dysregulation of BAs is linked to NASH pathogenesis; therefore, modulating BA homeostasis opens potential therapy of NASH through their signaling effects.^80^ The ratio of secondary BAs to primary BAs is inversely correlated to the NAFLD Activity Score, indicating a relationship between BA species and disease stage.^81^,^82^ Free fatty acid accumulation from diet inhibits *Shp* expression, leading to decreased repression of *Ntcp* and *Cyp7a1* and continued BA production and accumulation in the liver, which promotes hepatocyte injury and the development of NASH.^83^,^84^ Because of the close relationship between the microbiome and BA composition, it has been established that there are differences in microbiome composition between healthy and NAFLD patients. Glycine-metabolizing and taurine-metabolizing bacteria were increased in NAFLD patients, which may help explain the increase in secondary BAs in the BA pool.^85^ Furthermore, when intestinal microflora composition is altered, conjugated BAs and their metabolites can be increased, which inhibits intestinal FXR signaling leading to reduced BA secretion from the liver and promotion of NAFLD.^86^

#### Benefits of tissue-specific FXR activation/inactivation in the treatment of NASH

The current challenge is the design of tissue-specific FXR agonists capable of regulating BA homeostasis, lipid metabolism, and inflammation without off-target effects. Systemic FXR activation is proven to be beneficial in protecting against steatosis, inflammation, and fibrosis because of its activation of FXR. Systemic FXR agonists, such as obeticholic acid, reduce the accumulation of triglycerides in the liver and free fatty acids in mice fed high-fat diet.^11^ Obeticholic acid also decreases liver inflammation and fibrosis while increasing the risk of pruritus and LDLs.^8^,^87^ GW4064, a selective FXR agonist, has been shown to reduce hepatic inflammation in high-fat diet or high-fat, high-cholesterol diet–fed mouse models.^10^ WAY-362450 decreased fibrosis severity in methionine and choline-deficient mouse models^88^ and increased VLDL and LDL while decreasing HDL in fructose-fed rats.^89^ Cilofexor (GS-9674) is beneficial in decreasing steatosis and fibrosis in both mice and humans but increases the risk of pruritus.^90^,^91^ Tropifexor (LJN452) is also beneficial in decreasing liver fat and fibrosis while increasing the risk of pruritus and is associated with minor increases in LDLs.^9^ The benefits and consequences of whole-body FXR agonists demonstrate the importance of understanding mechanisms and/or roles of FXR tissue-specific activation to negate adverse effects in patients with liver diseases.

Genome-wide ChIP-seq technologies have allowed insight into tissue-specific gene expression of FXR in mice.^92^ There was only an 11% overlap between liver and intestinal FXR-binding sites in mice, suggesting underlying regulation of FXR tissue-specific functionality.^92^ Activation of hepatic FXR has been shown to be a protective mechanism against hepatic steatosis.^93^ It was determined that when feeding hepatic FXR knockout and intestinal FXR knockout mice a high-cholesterol diet, the hepatic FXR deficiency exacerbated hepatic steatosis while intestinal FXR deficiency did not.^93^ Hepatic FXR also inhibits lipogenesis by inducing SHP expression, which suppressed sterol regulatory element-binding protein 1c and its downstream lipogenic target genes.^94^ In addition, hepatic FXR is important in modulating hepatic inflammation, specifically by inhibiting NF-κB, an important inflammatory modulator.^7^ *In vitro*, FXR inhibited NF-κB activation in HepG2 cells and primary hepatocytes.^7^ *In vivo*, FXR knockout mice treated with LPS had greater induction of hepatic proinflammatory mediators, such as cyclooxygenase-2 and inducible nitric oxide synthase, compared with the control group, implicating an anti-inflammatory characteristic of hepatic FXR.

The activation and inhibition of intestinal FXR have been beneficial in the treatment of NASH in rodents. The benefits of the inhibition of intestinal FXR have been attributed to the microbiome-intestine-liver ceramide axis.^95^ Ceramides are intracellular signals for apoptosis^96^ and also increase sterol regulatory element-binding protein 1c activity in the liver, which promotes lipogenesis.^97^ Intestinal FXR has been shown to increase the expression of genes involved in ceramide synthesis.^86^,^97^ Mice fed a high-fat diet treated with a bile salt hydrolase inhibitor, caffeic acid phenethyl ester, displayed reduced intestinal FXR activity and ceramide synthesis. Treatment with caffeic acid phenethyl ester lowered average body weight and improved insulin sensitivity and glucose tolerance.^98^ The reduction in ceramide levels also reduced hepatic endoplasmic reticulum stress. It is also known that caffeic acid phenethyl ester activates the cAMP-CREB pathway, which may be the mode of action for bile salt hydrolase gene downregulation. Benefits of activating intestinal FXR, outside of BA synthesis regulation, include improvements in energy metabolism. Mice fed control or high-fat diet treated with fexaramine, an intestinal-specific FXR agonist, demonstrated increased energy expenditure, reduced body weight and body fat mass, decreased systemic inflammation and glucose production, and increased brown adipose tissue mass when compared with vehicle-treated mice on the high-fat diet.^99^ Because of the complex responses of tissue-restricted FXR activation, identifying tissue-specific or cell-specific modulators of FXR is required to develop safe and effective therapies for NAFL and NASH patients.

### Tissue specificity of NRs

#### TF complexes in NASH

It is widely accepted that TFs work in a complex network for the regulation of gene transcription and repression, which can become altered in diseased states.^100^,^101^ HNF4α is a well-studied TF highly enriched in the liver and is important for maintaining liver function and mature hepatocyte function. C57BL/6J mice overexpressing human HNF4α exhibited protective effects against diet-induced NASH, whereas loss of HNF4α displayed opposite effects.^102^ The explained mechanisms involve transcriptional regulation of BA, lipolytic, and p53 signaling pathways. Restoration of *HNF4α* through mRNA delivery improves the functionality of fibrotic primary hepatocytes isolated from mice and humans.^103^ HNF4α also interacts with other TFs to elicit liver protective effects. It has been shown that HNF4α is required for activating transcription factor 3 (ATF3)-associated improvement of steatohepatitis.^104^ Mice fed a high-fat diet displayed increased hepatic carbohydrate-responsive element-binding protein and the inclusion of fructose to a high-fat diet increases both carbohydrate-responsive element-binding protein and *Srbep1* expression.^105^ In addition, it has been found that zinc fingers and homeoboxes 2 (ZHX2), and its downstream target protein PTEN, are suppressed in murine models of NASH and in steatotic hepatocyte culture.^106^ Hepatocyte-specific deletion of ZHX2 exacerbated murine NASH phenotype, whereas hepatocyte-specific overexpression ameliorated hepatic steatosis, lipid accumulation, and liver fibrosis and inflammation through increased expression of PTEN.^106^ A case for cellular programming through TF regulation in NASH has also been speculated during fetal development.^107^ Pups born to female rats with 50% food restriction during pregnancy and nursed by control dams had reduced hepatic peroxisome proliferator-activated receptor (PPAR)–α and -γ until 9 months of age, which may indicate a complex and developmentally linked role for PFs and TFs in NASH development.^108^ Together, these studies allow for the speculation that FXR expression and function in NASH may be disrupted through protein complex dysregulation; however, the varied expression of FXR through the body, and lack of FXR proteome knowledge, make it a difficult target for study.

#### Therapeutics of tissue-specific NRs

Cell-specific modification of the functions of 1 NR, estrogen receptor (ER), has been successful in the development of efficacious and safer medicines in the tissue-specific treatment of diseases. ERs are type I NRs whose tissue specificity has allowed researchers to design cell-specific agonists and antagonists, which have been extensively reviewed.^109^,^110^ Three types of predominant ERs have been discovered and characterized: ERα, ERβ, and an estrogen G protein–coupled ER (GPER1) with 2 main signaling mechanisms, genomic and nongenomic.^109^ Studies of estrogens and ERs in cancer, like breast and ovarian, have provided seminal knowledge on targeted drug development of selective ER modulators (SERMs) and identification of xenoestrogens to control ER function in a tissue-specific manner.^109^,^110^ Tamoxifen is a widely used SERM that can serve as an ERα agonist in uterine tissue and antagonist in breast tissue for the treatment of patients with breast cancer.^109^ Tamoxifen exerts its inhibitory function in breast tissue by interacting with ERα to shift the side chain to block coactivator binding^111^; however, its weak activator function for uterine ERα has been shown to increase endometrial proliferation and carcinogenesis.^109^,^110^

Chronic liver diseases, like NASH, with no FDA-approved therapeutic treatment, have benefitted from liver-specific NR targeting like thyroid hormone receptor–β (TR-β).^112^ TR-β is a nonsteroidal type I NR with extensive effects on metabolism, including body weight and LDL reduction and increased hepatic fatty acid β-oxidation on activation by thyroid hormones.^113^ Selective modulation of TR-β in the liver by promising drugs such as resmetirom (MGL-3196) and VK2809 (MB07811) have shown to be beneficial in NASH patients participating in phase 2 studies. Resmetirom significantly reduced hepatic fat after 12 and 36 weeks of treatment in NASH patients, whereas VK2809 significantly reduced liver fat content in treated NAFLD patients compared with a placebo on 12 weeks of administration.^114^,^115^ The ability of these compounds to specifically activate the TR-β isoform in the liver is critical to minimize any potential off-target effects of TR-β agonism in the central nervous system and hypothalamic-pituitary-thyroid axis, as TR-β has been demonstrated to affect remyelination.^116^ In addition, activating liver-specific TR-β minimizes side effects that occur in the heart and bone, which express TR-α. Through the tissue-specific effects of ER and TR-β treatments, the existence of unique tissue-specific FXR function is a promising avenue to investigate pharmacological strategies that can be implemented in the treatment of NASH.

## SUMMARY

BAs are instrumental in fat and lipid digestion and in the activation of numerous metabolic pathways; however, their accumulation in tissues can lead to cell damage. FXR, the master regulator of BA homeostasis, is critical in suppressing BA synthesis by negative feedback pathways and promoting BA transport therefore decreasing the risk of developing cholestasis and liver injury. NASH is one of the most common liver diseases, and cholestasis contributes to NASH development and progression into irreversible ailments. The regulation of BAs has been a key therapeutic strategy to maintain a healthy state in NASH patients. Whole-body FXR agonism often results in adverse effects such as pruritus and elevated serum LDL. Because of its ubiquitous expression, understanding and manipulating cell-specific FXR function may be the key for developing NASH therapeutics. PFs such as FOXA2 provide a novel area of study that contributes to underlying mechanisms determining tissue-restricted FXR modulation. After previous studies on NRs like ERα and TR-β, we are hopeful that the discovery of the tissue-specific transcriptional function of FXR will allow us to examine the targeted therapeutic approaches for NASH and other liver diseases.
